# Prevalence of Bacterial Vaginosis and Its Association With Preterm Birth in a Tertiary Care Hospital in Chennai: A Cross-Sectional Study

**DOI:** 10.7759/cureus.57502

**Published:** 2024-04-03

**Authors:** Kalpana N, Amirtha C, Lavoanya P, Logeswari BM

**Affiliations:** 1 Obstetrics and Gynaecology, Tagore Medical College and Hospital, Chennai, IND; 2 Obstetrics and Gynaecology, Vinayaka Missions Medical College and Hospital, Karaikal, IND; 3 Obstetrics and Gynaecology, Sree Balaji Medical College and Hospital, Chennai, IND

**Keywords:** neonatal mortality, disease prevention and control, amsel’s criteria, preterm labour, bacterial vaginosis (bv)

## Abstract

Background

The most common preventive cause of premature labour is ascending infections. This study was conducted to evaluate the association between bacterial vaginosis (BV) and preterm labour in antenatal women and determine the significance of using the Amsel criteria to screen for BV.

Methods

This was a hospital-based cross-sectional study conducted among 100 antenatal mothers in the second trimester attending the antenatal OPD at a tertiary care hospital in Chennai from October 2019 to September 2021 after obtaining clearance from the institutional ethics committee and written informed consent from the study participants. Data were entered in Excel (Microsoft Corporation, Redmond, WA) and analysed in SPSS (IBM Corp., Armonk, NY).

Results

According to the Amsel criteria, BV was detected in 21 women (21%). Neither maternal age nor parity had an effect on the study group. There was a statistically significant relationship (p < 0.05) between the mode of delivery, preterm labour, and the study group. Of the 21 positive BV cases, 95% were positive for clue cells and only 5% were positive for gram-negative bacteria. Consequently, BV was found to be associated with early labour. There is no association between BMI and BV (p > 0.005).

Conclusion

In the current study, BV was shown to be associated with preterm labour. Our study underscores the significance of the Amsel criteria as a valuable tool for screening BV in antenatal women.

## Introduction

Preterm delivery is a major contributor to neonatal morbidity and mortality and its prevention has assumed special importance in the practice of obstetrics. It is a well-known fact that neonatal mortality and morbidity rise exponentially with decreasing gestational age [1]. Preterm labour is defined as parturition that happens between the weeks of 20 and 36 + 7 days of pregnancy. It is further divided into two categories: early preterm and late preterm. When a baby is born before 33 weeks + six days, it is considered early preterm, and when a baby is delivered between 34 weeks and 36 weeks + six days, it is considered late preterm. In India, 3.6 million babies are born prematurely each year, out of a total of 27 million. It is thought to be responsible for 40% of all newborn fatalities globally, and it affects one out of every 10 births each year (WHO, 2018). Preterm birth has long-term repercussions that extend from infancy to adolescence and adulthood [2].

Stress, infection, placental abruption, placenta previa, substance use, history of preterm birth or abortion, inadequate prenatal care, smoking, maternal age of <18 or >40 years, poor nutrition, low BMI, foetal anomaly, foetal growth restriction, oligohydramnios, polyhydramnios, vaginal bleeding, premature preterm rupture of membranes (PPROM), and environmental factors are some of the aetiologies that can cause preterm labour Prematurity is the major worry with PPROM. Respiratory distress is the most prevalent consequence of preterm delivery. However, sepsis, intraventricular haemorrhage, and necrotizing enterocolitis must also be considered. Early gestational age at membrane rupture is linked to an increased risk of newborn white matter injury, and PPROM with intrauterine inflammation can cause neurodevelopmental impairment [3]. Preterm birth is a global concern, with Africa and South Asia accounting for more than 60% of all preterm births. On average, 12% of babies are born prematurely in low-income nations, compared to 9% in high-income countries. India has 3,519,100 preterm births, followed by China, Nigeria, Pakistan, Indonesia, the USA, Bangladesh, the Philippines, the Democratic Republic of the Congo, and Brazil [4,5].

Cervical alterations, prolonged uterine contractions, and activation of the decidua and membranes all lead to labour. The difference between term and preterm labour is that the former is caused by a normal physiologic process, while the latter is caused by a pathological condition. Sometimes the process is quick, while others might take weeks to reach premature labour. Foetal fibronectin detection in cervicovaginal secretions is used to measure extracellular matrix disintegration, which is also a part of the parturition process. It shows a disturbance of the decidual-chorionic interface and an elevated risk of preterm labour when found between 22 and 37 weeks of pregnancy. Apoptosis appears to play a key role in the aforementioned process, according to evidence [6].

Infections are responsible for over 40% of premature labour cases. The most common preventive cause of premature labour is ascending infections. Bacterial vaginosis (BV) is a common cause of premature labour among ascending infections. In BV, anaerobic organisms replace the usual vaginal flora. *Gardnerella vaginalis*, *Bacteroides*, *Mobiluncus*, *Mycoplasma hominis*, *Peptostreptococcus*, *Fusobacterium*, and *Prevotella* are the species in question. The most frequent lower genital tract syndrome among women of reproductive age is BV. According to recent research, the incidence of BV among non-pregnant women ranges from 15% to 30%, while up to 50% of pregnant women have been diagnosed with BV. BV is diagnosed using a variety of techniques [7]. Amsel’s criteria, gram stain (Nugent score/Hay Ison grading), and the BV blue test, which analyses vaginal fluid sialidase activity, are some of the procedures used [8,9].

Amsel’s criteria are regularly used diagnostic criteria for BV. They entail evaluating four clinical characteristics and determining whether three or more of them correspond to a diagnosis of BV. For a confirmed diagnosis of BV, at least three of the four criteria must be met. Antibiotics, both systemic and topical, can heal BV, with spontaneous recurrence happening more frequently in women treated with topical antibiotics than in women treated with systemic antibiotics. This study also sought to identify the most important Amsel’s criteria that may be used alone to diagnose BV without jeopardising the diagnostic power of the entire Amsel's criteria set. This is especially important in developing nations like ours, where resources and time are limited. This study was done to estimate the association between BV and preterm labour in antenatal women and also to determine the significance of using Amsel criteria to screen for BV.

## Materials and methods

This was a hospital-based cross-sectional study conducted among 100 antenatal mothers in the second trimester attending the antenatal OPD at a tertiary care hospital in Chennai from October 2019 to September 2021 after obtaining clearance from the human institutional ethics committee (002/SBMC/IHEC/2019/1287) and written informed consent from the study participants.

The sample size was calculated to be 100, based on the formula 4 pq/L square and taking the allowable relative error as 10% and prevalence as 50% (based on a previous study done at Kyoto University, Japan in 2018) [10]. Universal sampling was adopted till sample size was achieved. The inclusion criteria included all antenatal mothers attending the antenatal OPD in the second trimester with the exclusion of the following conditions: incompetent cervical os, foetal anomalies, malformation of the uterus, polyhydramnios, and twin pregnancy.

A comprehensive medical history was collected, including menstrual and obstetrical history. The gestational age was determined based on the last menstrual cycle, clinical exams, and ultrasonographic gestational age. A complete history of pregnancy complications was obtained throughout the present pregnancy. A vaginal and speculum examination was performed on the abdomen after verbal and written consent. Vaginal swabs were obtained for bacteriologic testing once the nature of the discharge was noticed.

The patient was placed in a dorsal supine posture to collect the samples. The posterior vaginal wall was retracted using a simple speculum under complete aseptic precautions, and vaginal swabs were collected from the posterior fornix with sterile cotton swabs. The pH of the vaginal fluid was determined by using a piece of nitrazine paper. It is a sensitive test and over 90% of patients with BV have a pH of more than 5.

BV screening was done by the Amsel criteria, which states the diagnosis of BV was based on the presence of three or all four of the below-mentioned criteria, which include homogenous thin vaginal fluid that adheres to the vaginal wall, vaginal pH greater than 4.5, an amine odour when vaginal secretions are mixed with 10% potassium hydroxide (whiff test), and presence of clue cells in the vaginal discharge, seen in wet mount.

The study participants were categorised based on the Amsel criteria into two categories. Category A included mothers who tested positive for BV and were treated according to the CDC guidelines while category B included mothers who tested negative for BV.

Body mass index was calculated by obtaining their height and weight. BMI was categorised as <18.5 (underweight), 18.5-24.9 (normal), 25.0-29.9 (overweight), 30.0-34.9 (obese I), 35.0-39.9 (obese II), and ≥40.0 (obese III) based on the Asian-Pacific classification of obesity.

Statistical methods

The data were entered in Microsoft Excel (Microsoft Corporation, Redmond, WA) and analysed using SPSS version 26 (IBM Corp., Armonk, NY). All categorical variables were expressed as frequency with percentage. The association between categorical variables was done using a chi-square/Fischer exact test and continuous variables by unpaired t-test/Mann-Whitney test. A p-value less than 0.05 was considered to denote a significant relationship.

## Results

Of the total 100 participants, the mean ± SD age of the study participants was 26.2 ± 3.5 years. Socioeconomic status was assessed using the modified BG Prasad scale, where none of the patients in the study belonged to the socioeconomic class I (upper) and class V (lower). Class III (middle) accounted for 55 (55%) of the total participants, whereas class IV (lower middle) accounted for 35 (35%). About 52 (52%) women were multigravida and 48 (48%) were reported as primigravida. Among the total number of cases in the present study, 32% were born vaginally compared to 68% who were born by caesarean. Of the preterm neonates, 56% were born by C-section, compared to 12% of full-term babies (Table 1).

**Table 1:** Sociodemographic characteristics of the study population (N = 100) ^*^ Modified BG Prasad scale. ^# ^Asia-Pacific classification of BMI.

Characteristics		Frequency	Percentage
Age	Mean ± SD	26.2 ± 3.5	
Socioeconomic status^*^	Upper	0	0
	Upper middle	10	10
	Middle	55	55
	Lower middle	35	35
	Lower	0	0
Parity	Multigravida	52	52
	Primi	48	48
Mode of delivery	Normal vaginal delivery	32	32
	Caesarean section	68	68
Pre-term labour	No	85	85
	Yes	15	15
Co-morbidity	Anaemia	30	30
	Gestational diabetes	12	12
	None	58	58
Neonatal complications	Respiratory distress	23	23
	Neonatal hypoglycaemia	3	3
	None	74	74
BMI status^#^	Underweight	45	45
	Normal	30	30
	Overweight/obese	25	25

According to the Amsel criteria, BV was detected in 21 women (21%). Of the 21 positive BV cases, 95% were positive for clue cells and only 5% were positive for gram-negative bacteria. This implies that very few of the patients were severely infected.

Table 2 shows the distribution and association of BV and sociodemographic characteristics among study participants. The mean age of the participants with BV present was slightly higher at 26.5 years compared to those with BV absent at 26.1 years. There was a significant association between mode of delivery and BV presence, with nine (42.9%) BV present cases having been delivered through normal vaginal delivery. Pre-term labour was more common among participants with BV present (7, 33.3%) compared to those with BV absent (8, 10.2%). Consequently, BV was found to be associated with early labour.

**Table 2:** Distribution and association of bacterial vaginosis and sociodemographic characteristics among the study participants (N = 100) ^*^ Modified BG Prasad scale. ^#^ Asia-Pacific classification of BMI. BV: bacterial vaginosis.

Characteristics		BV absent (79), N (%)	BV present (21), N (%)	p-value
Age	Mean ± SD	26.1 ± 3.6	26.5 ± 3.2	0.44
Socioeconomic status^*^	Upper middle	8 (10.1)	2 (9.5)	0.94
	Middle	44 (55.6)	11 (52.4)	
	Lower middle	27 (34.3)	8 (38.1)	
Parity	Primi	38 (48.2)	10 (47.6)	0.54
	Multigravida	41 (51.8)	11 (52.4)	
Mode of delivery	Normal vaginal delivery	23 (29.1)	9 (42.9)	0.03
	Caesarean section	56 (70.9)	12 (57.1)	
Pre-term labour	No	71 (89.8)	14 (66.7)	0.01
	Yes	8 (10.2)	7 (33.3)	
Co-morbidity	Anaemia	23 (29.1)	7 (33.3)	0.7
	Gestational diabetes	9 (11.3)	3 (14.3)	
	None	47 (59.6)	11 (52.4)	
Neonatal complications	Respiratory distress	9 (11.3)	14 (66.7)	0.1
	Neonatal hypoglycaemia	2 (2.6)	1 (4.8)	
	None	68 (86.1)	6 (28.5)	
BMI status^#^	Underweight	34 (43.1)	11 (52.4)	0.2
	Normal	26 (32.8)	4 (19.1)	
	Overweight/obese	19 (24.1)	6 (28.5)	

Table 3 shows the distribution and association of BV and microbiological factors among study participants. All participants with BV had an acidic pH, with no one having an alkaline pH. The presence of clue cells and gram-negative bacteria cells was significantly associated with BV presence. A fishy odour on the whiff test was strongly associated with BV presence, with 20 (95.2%) of BV present cases having a fishy odour, whereas 78% of the total 79 negative BV cases showed no fishy odour. This was statistically significant when it was correlated with gram staining results, where four (5%) cases have gram-negative infections.

**Table 3:** Distribution and association of bacterial vaginosis and microbiological factors among the study participants (N = 100) BV: bacterial vaginosis; GNB: gram-negative bacteria.

Characteristics		Total	BV absent (79), N (%)	BV present (21), N (%)	p-value
pH	Acidic	100	79 (100)	21 (100)	<0.001
	Alkaline	0	0	0	
Whiff test	Fishy odour	21	1 (1.3)	20 (95.2)	<0.001
	No fishy odour	79	78 (98.7)	1 (4.8)	
Microscopy	Presence of clue cells	21	1 (1.3)	20 (95.2)	<0.001
	Presence of GNB cells	5	4 (5.1)	1 (4.8)	
	Absence of clue cells	72	72 (91.1)	0	
	Absence of GNB cells	2	2 (2.5)	0	

## Discussion

Premature delivery is a primary cause of newborn sickness and mortality. According to an increasing corpus of studies, infections appear to have a crucial role in causing preterm birth. BV was detected in 21% of preterm cases, according to Amsel's criteria in our study. Moreover, in 2000, the *American Family Physician* reported that 6,540 of the 21,965 women evaluated had BV, which is the same proportion as the current research [11].

Similar to the age distribution in our study, BV was most common in the age group of 18-27 years, and in primipara at the gestational ages of 11-20 weeks in a similar study [12], which also justifies women aged 25 to 34 years old had the greatest prevalence of BV and these women are also the most sexually active. The socioeconomic status is in line with the findings of a study conducted by the Department of Microbiology at the Government Medical College in Patiala, Punjab (2001) [13]. Contrary to the findings of this study, BV was shown to be frequent in Peruvian women from low-income neighbourhoods, with a prevalence of 27.0% [14].

Ibrahim et al. [15], on the other hand, discovered that the multigravida had the highest incidence, which contradicts our findings; however, the prevalence of BV was highest in the second trimester of pregnancy, which is consistent with our study findings but differs from Abdulameer's findings, which revealed that the prevalence of BV decreased as the trimester proceeded [16]. People with iron deficiency anaemia, cystitis, or BV are more likely to have preterm labour. According to Wilson (2017), pregnant mothers with iron deficiency anaemia had a four-fold higher risk of preterm labour (OR = 4.04, 95% CI = 1.15 to 14.16, p = 0.023) than those without iron deficiency anaemia [17].

Premature labour and early and late spontaneous abortions have all been linked to increased pH and/or BV in investigations. Hemalatha et al. [18] observed that 16% of women who only recognised BV infections were diagnosed at vaginal pH values over 4.5 as BV cases towards the end of the second trimester of pregnancy. Platz-Christensen et al. (1993) studied the vaginal pH and the persistence of clue cells in Papanicolaou-stained smears in 119 pregnant women, which showed the same results as ours [19]. The whiff test was positive in 95% of the 21 positive BV cases, which is also consistent with another study of 83.3% clue cells [20].

When evaluating multiple diagnostic techniques for BV, a study [21] discovered that when compared to the Nugent scoring system, Amsel's clinical criteria had a sensitivity of 85.7% and a specificity of 98.0%. The presence of clue cells was determined to be the most specific and sensitive individual Amsel's criterion.

Based on the significant association observed, it is recommended to incorporate routine screening for BV using Amsel criteria as a standard practice in antenatal care. This proactive approach may aid in early detection and timely intervention. Longitudinal studies are required to monitor the long-term effects of BV on maternal and neonatal health. This can provide insights into the persistence of the condition and its impact on subsequent pregnancies.

Limitations

The limitations of the study include generalizability, as the study was conducted in a tertiary care hospital in Chennai, which may not represent the broader population. Multi-centre studies involving different geographic locations and demographics would enhance the external validity of the results. The cross-sectional design limits the ability to establish causation.

## Conclusions

In conclusion, our study underscores the significance of the Amsel criteria as a valuable tool for screening BV in antenatal women. The observed association between BV and preterm labour highlights the potential role of proactive screening and intervention in mitigating adverse pregnancy outcomes. With 21% of antenatal women exhibiting BV according to the Amsel criteria, our findings emphasise the importance of incorporating routine screenings into antenatal care protocols. The statistically significant relationship between mode of delivery, preterm labour, and BV further emphasises the clinical relevance of identifying and addressing this condition.
